# Supplementary material for: The influence of lightweight wearable resistance on whole body coordination during sprint acceleration among Australian Rules football players
Source: PLoS One. 2024 Nov 5;19(11):e0313290. doi: 10.1371/journal.pone.0313290 (PMC11537414; doi:10.1371/journal.pone.0313290)
Supplement: S1 Table — (DOCX) [file pone.0313290.s001.docx]

|  |  | Baseline | HAT | LAT | HPT | LPT | HAS | LAS | HPS | LPS |
| --- | --- | --- | --- | --- | --- | --- | --- | --- | --- | --- |
| 10 m split (s) | P1 | 1.94 (1.91 – 1.96) | 1.97 (1.96 – 1.98) | 2.00 (1.95 – 2.03) | 1.97 (1.94 – 2.04) | 1.95 (1.92 – 2.01) | 1.97 (1.94 – 1.99) | 1.94 (1.91 – 1.96) | 1.97 (1.96 – 2.00) | 1.96 (1.95 – 1.97) |
|  | P2 | 2.12 (2.08 – 2.15) | 2.19 (2.12 – 2.27) | 2.25 (2.22 – 2.27) | 2.19 (2.14 – 2.24) | 2.15 (2.12 – 2.17) | 2.15 (2.11 – 2.19) | 2.16 (2.14 – 2.17) | 2.14 (2.12 – 2.15) | 2.20 (2.19 – 2.21) |
|  | P3 | 1.96 (1.93 – 2.01) | 1.99 (1.95 – 2.04) | 1.95 (1.92 – 1.97) | 1.91 (1.89 – 1.93) | 1.97 (1.95 – 1.99) | 1.97 (1.96 – 1.99) | 1.99 (1.94 – 2.04) | 2.01 (1.98 – 2.04) | 2.03 (2.00 – 2.09) |
|  | P4 | 1.89 (1.86 – 1.92) | 1.96 (1.94 – 1.99) | 1.91 (1.91 – 1.92) | 1.90 (1.88 – 1.91) | 1.91 (1.90 – 1.92) | 1.91 (1.89 – 1.94) | 1.92 (1.89 – 1.94) | 1.92 (1.91 – 1.93) | 1.90 (1.89 – 1.91) |
|  | P5 | 1.96 (1.92 – 1.98) | 1.97 (1.96 – 1.98) | 1.95 (1.94 – 1.96) | 2.01 (2.01 – 2.02) | 1.93 (1.92 – 1.94) | 1.90 (1.89 – 1.93) | 1.95 (1.93 – 1.96) | 1.94 (1.92 – 1.95) | 1.93 (1.88 – 1.98) |
|  | Group | 1.98 (1.86 – 2.15) | 2.02 (1.94 – 2.27) | 2.01 (1.91 – 2.27) | 2.00 (1.88 – 2.24) | 1.98 (1.90 – 2.17) | 1.98 (1.89 – 2.19) | 1.99 (1.89 – 2.17) | 2.00 (1.91 – 2.15) | 2.00 (1.88 – 2.21) |
| COM velocity at 4 m (m.s^-1^) | P1 | 5.65 (5.53 – 5.76) | 5.54 (5.52 – 5.56) | 5.61 (5.54 – 5.69) | 5.48 (5.35 – 5.58) | 5.72 (5.69 – 5.77) | 5.61 (5.51 – 5.67) | 5.70 (5.61 – 5.75) | 5.61 (5.49 – 5.68) | 5.57 (5.55 – 5.60) |
|  | P2 | 5.42 (5.33 – 5.47) | 5.36 (5.31 – 5.40) | 5.30 (5.21 – 5.38) | 5.28 (5.21 – 5.37) | 5.43 (5.36 – 5.47) | 5.38 (5.37 – 5.38) | 5.40 (5.39 – 5.41) | 5.25 (5.23 – 5.29) | 5.39 (5.35 – 5.43) |
|  | P3 | 5.80 (5.70 – 5.83) | 5.60 (5.51 – 5.69) | 5.83 (5.79 – 5.87) | 5.79 (5.68 – 5.87) | 5.78 (5.73 – 5.83) | 5.78 (5.71 – 5.87) | 5.72 (5.63 – 5.77) | 5.62 (5.54 – 5.72) | 5.61 (5.55 – 5.66) |
|  | P4 | 5.62 (5.55 – 5.67) | 5.39 (5.23 – 5.50) | 5.53 (5.51 – 5.55) | 5.55 (5.50 – 5.58) | 5.52 (5.45 – 5.62) | 5.56 (5.47 – 5.61) | 5.51 (5.45 – 5.63) | 5.52 (5.48 – 5.58) | 5.60 (5.57 – 5.67) |
|  | P5 | 5.43 (5.19 – 5.61) | 5.70 (5.68 – 5.72) | 5.23 (5.10 – 5.39) | 5.49 (5.47 – 5.51) | 5.81 (5.74 – 5.94) | 5.44 (5.39 – 5.52) | 5.25 (5.16 – 5.30) | 5.53 (5.35 – 5.67) | 5.92 (5.89.- 5.95) |
|  | Group | 5.59 (5.19 – 5.83) | 5.52 (5.23 – 5.72) | 5.50 (5.10 – 5.87) | 5.52 (5.21 – 5.87) | 5.65 (5.36 – 5.94) | 5.55 (5.37 – 5.87) | 5.52 (5.16 – 5.77) | 5.50 (5.23 – 5.72) | 5.60 (5.35 – 5.95) |
