# Supplementary material for: The influence of lightweight wearable resistance on whole body coordination during sprint acceleration among Australian Rules football players
Source: PLoS One. 2024 Nov 5;19(11):e0313290. doi: 10.1371/journal.pone.0313290 (PMC11537414; doi:10.1371/journal.pone.0313290)
Supplement: S2 Table — (DOCX) [file pone.0313290.s002.docx]

|  | **Baseline vs HPT** | | | **Baseline vs HAT** | | |
| --- | --- | --- | --- | --- | --- | --- |
| **Distance rank** | **Joint/segment** | **Plane of motion** | **Distance** | **Joint/segment** | **Plane of motion** | **Distance** |
| 1 | Pelvis | Sagittal | 8.52 | Stance shoulder | Transverse | 6.2 |
| 2 | Stance shoulder | Transverse | 7.1 | Swing shoulder | Transverse | 5.35 |
| 3 | Stance hip | Sagittal | 7.08 | Thorax | Sagittal | 5.15 |
| 4 | Swing hip | Sagittal | 6.88 | Stance shoulder | Sagittal | 5.14 |
| 5 | Stance shoulder | Sagittal | 5.1 | Swing shoulder | Sagittal | 5.09 |
| 6 | Thorax | Sagittal | 4.49 | Stance hip | Transverse | 4.73 |
| 7 | Swing shoulder | Transverse | 4.2 | Swing hip | Transverse | 4.45 |
| 8 | Swing hip | Transverse | 4.02 | Stance hip | Sagittal | 4.21 |
| 9 | Swing shoulder | Sagittal | 3.8 | Swing hip | Sagittal | 4.11 |
| 10 | Stance shoulder | Frontal | 3.71 | Pelvis | Sagittal | 3.72 |
| 11 | Stance hip | Transverse | 3.63 | Swing knee | Frontal | 3.58 |
| 12 | Swing ankle | Frontal | 3.28 | Stance knee | Frontal | 3.54 |
| 13 | Thorax | Transverse | 3.15 | Swing knee | Sagittal | 3.44 |
| 14 | Swing ankle | Sagittal | 2.79 | Stance shoulder | Frontal | 3.35 |
| 15 | Stance ankle | Frontal | 2.72 | Swing ankle | Frontal | 3.32 |
| 16 | Stance hip | Frontal | 2.63 | Stance knee | Sagittal | 3.14 |
| 17 | Stance knee | Sagittal | 2.57 | Stance hip | Frontal | 3.11 |
| 18 | Stance ankle | Sagittal | 2.55 | Thorax | Transverse | 2.92 |
| 19 | Pelvis | Transverse | 2.44 | Swing knee | Transverse | 2.82 |
| 20 | Swing knee | Sagittal | 2.41 | Swing hip | Frontal | 2.77 |
| 21 | Swing knee | Frontal | 2.41 | Swing ankle | Sagittal | 2.65 |
| 22 | Swing shoulder | Frontal | 2.19 | Stance ankle | Sagittal | 2.39 |
| 23 | Stance ankle | Transverse | 2.14 | Swing shoulder | Frontal | 2.38 |
| 24 | Stance knee | Frontal | 2.12 | Swing ankle | Transverse | 2.37 |
| 25 | Swing hip | Frontal | 2.11 | Pelvis | Frontal | 2.35 |
| 26 | Swing ankle | Transverse | 1.94 | Pelvis | Transverse | 2.34 |
| 27 | Stance knee | Transverse | 1.85 | Stance ankle | Frontal | 2.23 |
| 28 | Pelvis | Frontal | 1.77 | Stance knee | Transverse | 2.19 |
| 29 | Swing knee | Transverse | 1.72 | Stance ankle | Transverse | 1.97 |
| 30 | Thorax | Frontal | 1.71 | Thorax | Frontal | 1.97 |
