# Supplementary figures and images for: The influence of lightweight wearable resistance on whole body coordination during sprint acceleration among Australian Rules football players
Source: PLoS One. 2024 Nov 5;19(11):e0313290. doi: 10.1371/journal.pone.0313290 (PMC11537414; doi:10.1371/journal.pone.0313290)

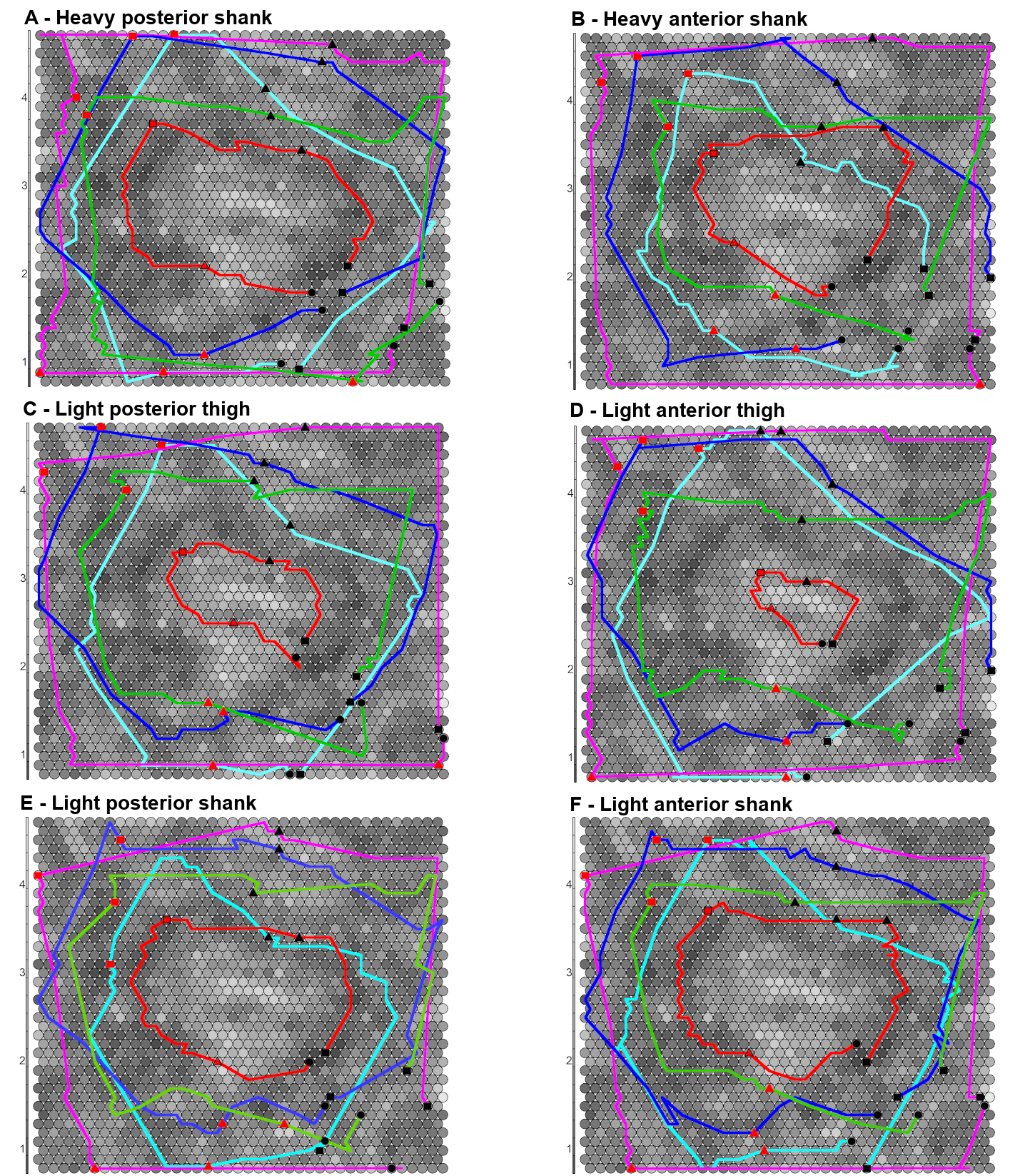

Supplement: S1 Fig — (A) Heavy posterior shank condition. (B) Heavy anterior shank condition. (C) Light posterior thigh condition. (D) Light anterior thigh condition. (E) Light posterior shank condition. (F) Light anterior shank condition. Participants are indicated by unique colours (light blue, P1; magenta, P2; dark blue, P3; red, P4; green, P5). Shapes and colours are used to indicate key phases of the stride cycle. Black circle, first left foot toe-off (beginning of stride) (TO1); red triangle, right foot touchdown (TD); red square, right foot toe-off (TO); black triangle, left foot touchdown (TD); black square, second left foot toe-off (end of stride) (TO2). (TIFF) [file pone.0313290.s003.tiff]
