# Supplementary material for: The influence of lightweight wearable resistance on whole body coordination during sprint acceleration among Australian Rules football players
Source: PLoS One. 2024 Nov 5;19(11):e0313290. doi: 10.1371/journal.pone.0313290 (PMC11537414; doi:10.1371/journal.pone.0313290)
Supplement: Attachment — Submitted filename: PONE_review_07-01-2024.pdf [file pone.0313290.s005.pdf]

Thank you for the opportunity to review "The influence of lightweight wearable resistance on whole body coordination during spring acceleration among Australian Rules football players". I was not a part of the first review and my comments reflect the manuscript as currently constituted. The sample is limited, and it is difficult to generalize to the entire population of semi-pro Australian Rules football players, or to similar sports, yet it is analyzing a newer form of training. Also, it is using analyses that can be done for group or individual level comparisons. I do have some major and minor comments which need to be addressed, whether it be changes to the manuscript text and/or reviewer responses.

Major Comments

Introduction:

The introduction is good, overall. In lines 65-68, I recommend expanding your example to be biomechanically more accurate. I assume you mean the orientation of the 2D (or 3D) resultant GRF, which is influenced by the planes being measured, therefore, the AP GRF has largest effect on that orientation. If possible, spell it out more clearly.

You need to refer to adaptability and performance in the introduction.

Methods:

Study Design:

At least 8 weeks to perform all of the testing from familiarization through the WR conditions is a long time. Are there any fatigue effects as the season progresses? I see the control with being at least 48 hours post-match or team training but is there an accumulation from the season? Without WR as an influence, do players maintain the same performance with sprinting speed and acceleration as the season progresses?

Consider including details about the warmup that was provided or allowed prior to the maximum sprints in this section rather than Data collection.

I thought of this while reading the discussion, why wasn't there a washout sprint. It would be interesting to see how the WR trials differ from sprint 1 without WR, and then in a washout (sprint 5), if some of the WR effects held.

Wearable resistance:

Did you measure the participants mass at each session? With such a large timespan, mass could change.

Did you determine a specific location on the thigh and shank to be consistent across sessions within participants, and the same relative location on a segment (e.g., middle third of shank) to be consistent across participants? I only ask because moment inertia involves more than the mass, distance is important to note as well.

Data collection:

Which dynamic mobility drills? Do you have a reference to indicate which drills and technique for them?

Who placed the markers on the participants, same person? Has that person been test for intra-session reliability on marker placements? If done by multiple people, has there been any inter-testing reliability performed?

Good work explaining the clustering analysis, SOM, and distance matrix. I think the explanations are clear.

Results:

The results are word-heavy because of the way it was analyzed and the small sample. Consider adding ranges to the data for Table 2 set to go along with means and SD, or range instead of SD because SD does not tell us as much with a small sample.

Discussion:

Lines 466-469: I can understand the desire to promote greater hip extension, but why would an athlete want transverse plane shoulder motion? I know they are examples based on the results, but maybe note that some motions are not wanted necessarily and overall, some motions being explored are not necessarily optimal.

Lines 476-483: How do you propose this individual level analysis? Also, to be ecologically driven, wouldn't it be best to analyze on the field during practice and require some type of sensor, such as an IMU?

The information from Lines 503-508 needs to be woven into the introduction as well. It will help drive home the point that adaptability is good and leads to higher performance.

Consider making mention of adaptability in the context of injury prevention, not just performance.

In the limitations section, make mention of the study not being ecological valid because data was collected in a laboratory, not on the playing field in a game or practice environment.

Conclusions

Good section, clean advice at the end.

Notes on Figures

Beautiful figures overall. One note, please bold the axes units and titles for figure 7 (joint kinematic data).

Minor Comments

Introduction:

Line 74: "…often utilized deconstructed, part practice drills..." Something is missing, or the punctuation is in the wrong place. Or deconstructed what?

Line 79-80: "…by coaches to afford specific movement…", consider changing "afford" to "influence".

Line 81: "Both approaches" should be spelled out because I am not sure which two approaches you are referring to. I am guessing deconstructed and constraint, but I am certain.

Line 88: Remove "particular" because the segments are given after the comma.

Line 91: Remove "particular"

Line 92: "…are seen as being indicative of overload having occurred []." Consider changing to "…indicate overload has occurred []."

Methods:

Line 114: Include the ".0" for "72" to stay consistent with the other means.

Lines 118-120: How was that average distance and average running speed determined for these players?

Line 221: Add "cubic" in front of "spline" which I assume was the polynomial spline used.

Lines 228-229: Indicate if a researcher identified these points or if the V3D kinematic algorithm did.

Results:

Lines 361-362: "…, except for in the heavy anterior shank condition…" does not fit with the sentence and should be deleted.

Line 439: Include a comma between "condition pelvic"

Discussion:

Lines 506-515: Condense this section, it is superfluous, especially the two sentences from 511-515.
