# Supplementary material for: The influence of lightweight wearable resistance on whole body coordination during sprint acceleration among Australian Rules football players
Source: PLoS One. 2024 Nov 5;19(11):e0313290. doi: 10.1371/journal.pone.0313290 (PMC11537414; doi:10.1371/journal.pone.0313290)
Supplement: Attachment — Submitted filename: Response to reviewers.docx [file pone.0313290.s004.docx]

**Response to reviewers**

Dear Dr. Arslan,

We thank you for the opportunity to revise our manuscript entitled “The influence of lightweight wearable resistance on whole body coordination during sprint acceleration among Australian Rules football players”. We would also like to thank the referees for their insightful feedback and suggestions. All reviewer comments are included in the document below in red coloured text, with our responses provided following each comment in black text. In the revised manuscript, we have primarily sought to focus the key messages of the paper. This has been achieved by rewriting and shortening sections of the manuscript to remove unnecessary depth and discussions that are not closely related to the study’s primary aim and findings. We have also made necessary formatting changes, addressed copywrite images, and included additional methodological detail where requested. We feel the quality of the revised manuscript is substantially improved.

Best regards,

Karl Trounson

Response to editor’s comments:

1. Please ensure that your manuscript meets PLOS ONE's style requirements, including those for file naming. The PLOS ONE style templates can be found at

https://journals.plos.org/plosone/s/file?id=wjVg/PLOSOne_formatting_sample_main_body.pdf and

https://journals.plos.org/plosone/s/file?id=ba62/PLOSOne_formatting_sample_title_authors_affiliations.pdf

We amended the file names of all figures to exclude spaces, such that these now read “Fig1.tif”.

We amended table titles to bold type throughout the manuscript.

Symbols for contributorship are now included on the title page.

Font and line spacing in references section has been amended according to style requirements.

We hope that these changes satisfy the style requirements as described in the referred templates.

2. Please include your full ethics statement in the ‘Methods’ section of your manuscript file. In your statement, please include the full name of the IRB or ethics committee who approved or waived your study, as well as whether or not you obtained informed written or verbal consent. If consent was waived for your study, please include this information in your statement as well.

An Ethics statement is included in the “Methods” section under the sub-section entitled “Participants”. Excerpts from the original manuscript read, “All [participants] provided written informed consent…All procedures used in this study complied with the criteria of the declaration of Helsinki and the ethical approval [was] granted by the Victoria University Human Research Ethics Committee.”

This statement includes the full name of the ethics committee who approved the study and states that written consent was obtained from participants.

Please advise if a separate statement under a distinct sub-section is required.

3. We note that Figure 1, 2, 3 and 4 in your submission contain copyrighted images. All PLOS content is published under the Creative Commons Attribution License (CC BY 4.0), which means that the manuscript, images, and Supporting Information files will be freely available online, and any third party is permitted to access, download, copy, distribute, and use these materials in any way, even commercially, with proper attribution. For more information, see our copyright guidelines: <http://journals.plos.org/plosone/s/licenses-and-copyright>.
 
We require you to either (1) present written permission from the copyright holder to publish these figures specifically under the CC BY 4.0 license, or (2) remove the figures from your submission:

a. You may seek permission from the original copyright holder of Figure 1, 2, 3 and 4 to publish the content specifically under the CC BY 4.0 license.

We recommend that you contact the original copyright holder with the Content Permission Form (<http://journals.plos.org/plosone/s/file?id=7c09/content-permission-form.pdf>) and the following text:
“I request permission for the open-access journal PLOS ONE to publish XXX under the Creative Commons Attribution License (CCAL) CC BY 4.0 (<http://creativecommons.org/licenses/by/4.0/>). Please be aware that this license allows unrestricted use and distribution, even commercially, by third parties. Please reply and provide explicit written permission to publish XXX under a CC BY license and complete the attached form.”

Please upload the completed Content Permission Form or other proof of granted permissions as an ""Other"" file with your submission.

In the figure caption of the copyrighted figure, please include the following text: “Reprinted from [ref] under a CC BY license, with permission from [name of publisher], original copyright [original copyright year].”

b. If you are unable to obtain permission from the original copyright holder to publish these figures under the CC BY 4.0 license or if the copyright holder’s requirements are incompatible with the CC BY 4.0 license, please either i) remove the figure or ii) supply a replacement figure that complies with the CC BY 4.0 license. Please check copyright information on all replacement figures and update the figure caption with source information. If applicable, please specify in the figure caption text when a figure is similar but not identical to the original image and is therefore for illustrative purposes only.

Figure 1 and 2 were originally produced by lead author Karl Trounson and have been previously published in PLoS ONE. As such, a request for permission form signed by Karl Trounson is now included in the submission, and the following attribution is provided in figure legends: “Reprinted from Trounson KM, Busch A, French Collier N, Robertson S (2020) Effects of acute wearable resistance loading on overground running lower body kinematics. PLoS ONE 15(12): e0244361 under a CC BY license, with permission from PLoS ONE, original copyright 2020.”

Figure 3 and 4 have been amended using an outline available in the public domain.

Responses to reviewer #1:

1-3 – Please revise the title, author´s name, and affiliation format, considering the journal template and instructions for authors.

Symbols for author contributorship are now included on the title page in line with formatting guidelines. Besides this, formatting of the title, authors’ names, and affiliations in the original manuscript appear to adhere to the journal requirements as set forth in the affiliations formatting guidelines: ([https://journals.plos.org/plosone/
s/file?id=3fac/PLOS Affiliations Formatting Guidelines.pdf](https://journals.plos.org/plosone/s/file?id=3fac/PLOS%20Affiliations%20Formatting%20Guidelines.pdf))

26 – Please write in full all abbreviations in the first appearance in the manuscript.

We have amended abbreviations throughout the manuscript in line with this feedback such that all are written out in full at first appearance.

21-45 – The abstract is too long, and results are missing. Moreover, no keywords are observed. Please revise.

The abstract in the original manuscript does not exceed 300 words, keeping in line with PLoS ONE submission guidelines. Data analysis in this study takes the form of cluster analysis, self-organising maps, and distance matrices, the results of which are described in lines 38-43 of the revised abstract. No inferential statistics were performed given the small sample size, which is why numerical data aren’t reported here.

There is no stipulation in submission guidelines to include keywords in the manuscript. These are provided during the submission process and can be seen on the first page of the submission file.

52 – Please revise the citation format.

PLoS citation style file for Endnote (available at: [https://endnote.com/downloads/
styles/plos-public-library-of-science-all-journals/](https://endnote.com/downloads/styles/plos-public-library-of-science-all-journals/)) was used for all references in the original manuscript. As such, all references should, and indeed appear to, adhere to the PLoS style requirements.

54 – Please revise the citations format (space between numbers). Please consider this in all manuscript.

Spacing between numbers if two consecutive citations are included appears to be the standard for PLoS articles. Again, the PLoS citation style file was used for referencing throughout the manuscript, and we understand that this is consistent with journal requirements.

63 – Too many citations. Revision is suggested.

Edits have been made throughout the manuscript to reduce the number of citations and omit less relevant information in order to keep the key points of the paper more clear. The number of citations is substantially reduced in the revised manuscript.

73-99 – Please consider shorter paragraphs in all manuscript to increase readability.

We acknowledge that lengthy sentences and paragraphs affect readability of the original manuscript and have made attempts to shorten these throughout in the revised manuscript. Much of the section identified by the reviewer (73-99) has been omitted or rewritten to improve clarity.

100 – Please correct the citation format. Same in line 243 (please revise all manuscript).

Citation formatting adheres to the PLoS citation style, in line with previous comment responses. In the specific line identified, the paper author is explicitly named in the text. We were unable to find formatting instructions for this type of instance, however, we have followed the format demonstrated in a recently published PLoS paper (<https://journals.plos.org/plosone/article?id=10.1371/journal.pone.0296860> - introduction section; paragraph 2; line 1).

Near 129 – The research gap and study aim should be clearly presented at the end of the discussion section.

The research gap and aim of the study are described in the final paragraph of the introduction. Edits have been made to this section to make the existing research gap into wearable resistance and the aim of the study clearer.

137 – Please indicate inclusion and exclusion criteria and describe the sample (performance level, training routines, and others).

We have made amendments to more clearly state inclusion and exclusion criteria. In addition, further information about typical Australian Rules football training habits of participants has been provided in the form of session frequency and typical running loads across a week.

142 – Please indicate the number of code approval.

Victoria University Human Research Ethics Committee code approval number (HRE19-020) has now been included in this section of the manuscript.

148 – Please indicate all procedures. Time of data collection? Circadian effect? Lab conditions (space, temperature, humidity). All procedures should be clearly understood by the readers.

Edits to the study design section have been made to include information relating to the time of data collection and laboratory conditions, as suggested.

222 – At the end of this section all details related to the methodology should be clearly understood by the readers. Please confirm.

Attempts have been made to clarify the details of the methodology in line with reviewer suggestions (e.g. laboratory conditions, participant training characteristics, testing session timing, and computation of joint kinematics). We recognise that details of data processing and data analysis may be challenging for readers without a biomechanics background, however, we have aimed to include as much detail as possible in order for the experimental setup and analyses to be reproduced.

271 – Please consider reorganizing this section aiming for more direct and clear information to the readers. For example, a sample of 5 is not mentioned. Sample Power (Gpower)? Additionally, the statistical analysis requires a more depth analysis, one M ± SD is observable.

This section has been amended to include the sample number included in the group average. The non-use of inferential statistics is emphasised throughout the manuscript and as such a power calculation is not relevant. The descriptive statistics form only a minor portion of the data analysis and are primarily reported for comparison with existing literature on the effects of WR during sprint acceleration. Hierarchical agglomerative clustering and self-organising maps serve as the main substance of the data analysis and we feel strongly that these are far more insightful analyses in relation to characterisation of whole body coordination changes in response to WR.

356 – Please consider the journal template and instructions for authors in the format of the table. Moreover, please revise the table content.

The formatting of the table has been corrected and the content revised to include a single main performance measure (20 m sprint time). 10 m split time and centre of mass velocity at 4 m have been moved to a supplementary table (S1 Table) to aid with interpretation and clarity of Table 2.

348-455 – Please consider a different format in the results section. Too many text difficult the interpretation.

The discussion has been restructured to aid with interpretation (see below), however, the results section is in convention with normal formatting, so this has been largely retained. We appreciate that there may be more text than normal, however, we feel that this is necessary in pointing the reader to pertinent elements of the hierarchical clustering dendrogram and self-organising map figures, which are the key elements of the results section.

455-594 - Please consider a different format in the discussion section (for example with subsections). Too many text difficult the interpretation.

Subsections have now been included in the discussion to aid interpretation and readability. In addition, the text has been shortened by removing unnecessary depth of commentary into proposed mechanisms of the observations. We hope that the revised version is more readable and interpretable and that key messages come across more clearly.

Near 594 - Please include suggestions for future research.

We suggest that future research should focus specifically on the effect of heavy anterior and posterior thigh wearable resistance on sagittal plane pelvis and hip kinematics among a larger athlete sample (lines 564 and 592).

538 - All references should be carefully revised, they are not according to the journal template and instructions for authors.

No reference was included in the line identified (538). Again, we would dispute that references are not formatted according to the journal template and instructions for authors, since the PLoS citation style file for Endnote was specifically used. We welcome specific examples from the reviewer in which we have deviated from the required citation style.

598-610 – Please consider more direct messages in the conclusions and end of the abstract. If possible, with practical application.

We have made amendments to the abstract and throughout the manuscript to focus the messaging and practical applications of the paper.

612 – More information is requested besides acknowledgments.

We are unsure what specific additional information is requested here. A financial disclosure statement and competing interests statement are provided on pages 1 and 2 of the submission file.

616 - Please revise the format of the manuscript considering the journal template and instructions for authors. Please check all details.

PLoS submission guidelines (<https://journals.plos.org/plosone/s/submission-guidelines>) have been closely reviewed and we are confident that the manuscript formatting adheres appropriately to these guidelines.

Please carefully revise English details throughout the manuscript.

Spelling, grammar and diction has been reviewed throughout the manuscript to ensure there are no errors and to clarify key messages of the paper.

Pages 43-49 – Please improve the figures quality. Please include the units in the axis.

Higher resolution versions of each figure are accessible through the hyperlinks at the top right corner of each figure page. All figures in the original submission meet the specified guidelines for quality in terms of dimensions and resolution (300 – 600 dpi). Although not always seen in self-organising maps, a label for the axis has now been included to clearly indicate that shading denotes a distance measure of arbitrary units between neighbouring points.

Responses to reviewer #2:

I thank the Authors and the Editors for the chance to review the present article. The manuscript investigates the influence of lightweight wearable resistance on whole-body coordination during sprints in Australian Rules football players. The manuscript is well structured and written, and I compliment the Authors for designing such a remarkable analysis. There are, however, some criticisms that should be addressed before the article can be deemed suitable for publication.

The introduction is informative and comprehensive about the topic. However, some paragraphs seem to be redundant, with the risk of making the section wordy and complicated to follow for the reader (e.g., lines 73-99). I would suggest streamlining the section and focusing more on the study rationale, which seems to be vague at this point. The purpose appears more in the direction of highlighting the possibility of the presented analysis framework being implemented in future larger studies rather than revealing possible effects in the presented dataset. It should be clarified whether the presented manuscript should be considered an original study, even if no inferential statistics have been conducted, from which to draw some conclusions or a framework to develop future studies.

We recognise that the length of the introduction negatively impacts the clarity of the study rationale and purpose. As such, superfluous information has been omitted (particularly in the passage identified by the reviewer) and other sections rewritten to improve clarity and readability. It has been clarified that the primary aim of the study is as an original study, which begins to direct coaches in applied settings interested in using WR for the purpose of sprint acceleration skill acquisition.

Similar to the introduction section, the discussion is way too extensive, and it seems to point the attention more on being a foundation for future studies (lines 460 and 469) rather than on the actual findings. I would suggest reorganizing the section, focusing more on the effects of WR. On a related note, the number of references (more than 100) seems excessive for a cross-sectional original study involving a small sample size; I would suggest reducing them in order to facilitate the readers to be informed on the topic.

In line with feedback about the length of the introduction section and comments regarding focussing of the primary study aim, appropriate amendments have also been made to the discussion. The amended section includes subheadings to direct the readier, avoids repetition of messaging around implication for future studies, and focusses on the consequential findings from the data. In addition, unnecessary depth of commentary into proposed mechanisms of the observations has been removed. We hope that these changes focus the reader’s attention on the findings related to the primary study aim, and that the possible mechanisms of changes and implications for coaching practice are more clear.

Minor comments:

Line 24: WR should be defined here as an acronym.

This has been amended to avoid use of the “WR” acronym in the abstract. “WR” is now defined at first appearance in the introduction section.

Line 58: a brief definition of the factors most influencing sprint acceleration could be helpful.

This sentence has been rewritten to include explicit mention of key factors influencing sprint acceleration; namely horizontal and vertical ground reaction impulse, and body segment positions. Subsequent lines have been rewritten to avoid redundancy of this message.

Line 64: please define the concept of “technique interventions”.

Wording has been amended here to clarify that this concept pertains to training interventions/drills aimed at improving sprint technique.

Lines 112-114: please clarify this notion.

This sentence refers back to ideas presented in paragraph 3 about modern skill acquisition perspectives, which view constraints on the movement system as control parameters that direct movement expression. This link may not be clear since the sentence in question appears somewhat later in the introduction. As such, the language in the sentence has been simplified to communicate the main point that WR has not been investigated for its potential use as a coaching tool to shape movement. We hope that this distinction is now more clear.

Line 135: what is the usual weekly/monthly training load of the involved athletes? What is their Australian Rules football experience?

More information has been provided about average participant weekly Australian Rules football training loads and structure to better contextualise results.

Lines 250 and 261: how were kinematics (CoM and angles) computed in terms of software/coding/modeling?

Spatiotemporal kinematics and joint angles were calculated using the purpose-built model based algorithms in Visual 3D software following tracking marker labelling and construction of 10-segment models for each participant. We have sought to amend the text to clarify that Visual 3D was used in these steps. Visual 3D provides extensive explanations of their model based algorithms on the C-Motion.com wiki page (<https://www.c-motion.com/v3dwiki/index.php?title=Main_Page>) if readers wish to explore this aspect further. As these algorithms are commonly used in biomechanical analyses, we feel that provision of their specific details is beyond the scope of this paper.

Lines 492-494: were inferential statistics used to study these differences?

Inferential statistics were not used to study these differences, again due to small sample size. The statement in this line refers to the small magnitude of difference on average in sprint acceleration outcome measures across conditions. We have sought to clarify this point in the revised manuscript.
