# Supplementary material for: The influence of lightweight wearable resistance on whole body coordination during sprint acceleration among Australian Rules football players
Source: PLoS One. 2024 Nov 5;19(11):e0313290. doi: 10.1371/journal.pone.0313290 (PMC11537414; doi:10.1371/journal.pone.0313290)
Supplement: Attachment — Submitted filename: Response to Reviewers.docx [file pone.0313290.s006.docx]

**Response to reviewers**

Dear Dr. Furlong,

We thank you again for the opportunity to re-revise our manuscript entitled “The influence of lightweight wearable resistance on whole body coordination during sprint acceleration among Australian Rules football players”. We would also like to thank Reviewer #2 for their support for the revised manuscript and Reviewer #3 for their insightful feedback and suggestions. Reviewer comments are included in the document below in red coloured text, with our responses provided following each comment in black text. In the revised manuscript, adjustments to the methods and study design sections have been made to include further details of warm-up procedures and WR placement. Details of the benefits of adaptability for field-based athletes have been included in the introduction and discussion sections. Finally, additional limitations pertaining to ecological validity and potential training adaptations across the 8 week testing period have been included. We thank the reviewer for their considered feedback and feel the quality of the revised manuscript is substantially improved.

Best regards,

Karl Trounson

Responses to Reviewer #3:

Reviewer #3: Thank you for the opportunity to review “The influence of lightweight wearable resistance on whole body coordination during spring acceleration among Australian Rules football players”. I was not a part of the first review and my comments reflect the manuscript as currently constituted. The sample is limited, and it is difficult to generalize to the entire population of semi-pro Australian Rules football players, or to similar sports, yet it is analyzing a newer form of training. Also, it is using analyses that can be done for group or individual level comparisons. I do have some major and minor comments which need to be addressed, whether it be changes to the manuscript text and/or reviewer responses.

Major Comments

Introduction:
The introduction is good, overall. In lines 65-68, I recommend expanding your example to be biomechanically more accurate. I assume you mean the orientation of the 2D (or 3D) resultant GRF, which is influenced by the planes being measured, therefore, the AP GRF has largest effect on that orientation. If possible, spell it out more clearly.

We have amended the point highlighted to clarify that the anteroposterior component of GRFs resulting from foot strike is where meaningful differences between faster and slower athletes are observed. The revised version includes greater biomechanical detail to remove ambiguity associated with the original term ‘forward ground reaction forces’.

You need to refer to adaptability and performance in the introduction.

We have amended the introduction to make mention of adaptability and its utility in maintaining performance for field-based athletes under dynamic and varying constraints.

Methods:
Study Design: At least 8 weeks to perform all of the testing from familiarization through the WR conditions is a long time. Are there any fatigue effects as the season progresses? I see the control with being at least 48 hours post-match or team training but is there an accumulation from the season? Without WR as an influence, do players maintain the same performance with sprinting speed and acceleration as the season progresses?

It does not appear that there is a fatigue effect in relation to maximal speed performance across the course of several weeks in-season. In Australian Football, no clear trend is apparent in relation to distance covered above 90% of maximum velocity across an 8 week period [1]. Week-to-week changes appear to be match specific (presumably resulting from differences in weather conditions, tactics, ground dimensions, etc.) Among soccer players, however, repeated measures testing of 15 m [2], 35 m [3], and 50 m [4] sprints over the course of a competitive period consistently show fastest sprint times exhibited at the end of the season. This is likely due primarily to the frequency of exposure to sprint efforts in season, as well as possibly decreases in body fat across the course of a season. We have included an acknowledgement of this exposure effect as a potentially confounding factor in our limitations section.

Consider including details about the warmup that was provided or allowed prior to the maximum sprints in this section rather than Data collection.

Details of the warm-up have been moved to the study design section in line with the reviewer’s suggestion.

I thought of this while reading the discussion, why wasn’t there a washout sprint. It would be interesting to see how the WR trials differ from sprint 1 without WR, and then in a washout (sprint 5), if some of the WR effects held.

We wholly concur that this represents a worthwhile area of further exploration. Findings from weighted bat swinging in baseball suggest that temporal sequencing remains acutely affected when batters return to a normal weighted implement [5, 6]. It is likely that some movement after-effects manifest in acceleration and it would be of interest to know whether these are in the direction of, or opposed to, the coordination patterns exhibited with the WR. We believe, however, that this specific question warrants standalone research and would be too extensive to include in the present investigation.

Wearable resistance: Did you measure the participants mass at each session? With such a large timespan, mass could change.

Participant body mass was measured at the commencement of each session. Based on moment of inertia calculations and the smallest WR fraction (50 g), fluctuations of approximately 2 kg were needed for a 180 cm tall participant to warrant altering the loading magnitude in the heavy thigh conditions. In light thigh conditions and shank conditions larger magnitude body mass changes were required. In each of the relevant testing sessions, there were no fluctuations of sufficient magnitude to warrant alteration of the initial prescribed WR load.

Did you determine a specific location on the thigh and shank to be consistent across sessions within participants, and the same relative location on a segment (e.g., middle third of shank) to be consistent across participants? I only ask because moment inertia involves more than the mass, distance is important to note as well.

Yes, WR loads were situated with the middle of the fusiform shape placed at the midpoint of the relevant segment. We have sought to clarify this in the methods.

Data collection: Which dynamic mobility drills? Do you have a reference to indicate which drills and technique for them?

The dynamic mobility drills implemented have now been specified and a reference provided describing the technical execution of each drill.

Who placed the markers on the participants, same person? Has that person been test for intra-session reliability on marker placements? If done by multiple people, has there been any inter-testing reliability performed?

The same experienced biomechanics lab technician applied markers to participants throughout the study. While we did not undertake inter-session reliability testing, this data could be calculated among the participant group for some or all markers if deemed necessary. Given the capability of the technician to identify anatomical landmarks and the athletic physique of participants, consequential between-session variation in marker placement was/is not expected.

Good work explaining the clustering analysis, SOM, and distance matrix. I think the explanations are clear.

We thank the reviewer for their positive feedback on this section.

Results:
The results are word-heavy because of the way it was analyzed and the small sample. Consider adding ranges to the data for Table 2 set to go along with means and SD, or range instead of SD because SD does not tell us as much with a small sample.

Table 2 and S1 Table have been amended to report the data means and ranges in line with the reviewer’s suggestion.

Discussion:
Lines 466-469: I can understand the desire to promote greater hip extension, but why would an athlete want transverse plane shoulder motion? I know they are examples based on the results, but maybe note that some motions are not wanted necessarily and overall, some motions being explored are not necessarily optimal.

For early sprint acceleration, frontal and transverse plane motion have been identified as important contributors to shoulder, thoracic, and hip peak angular velocity [7]. Increases to the lever arm of the arm and leg through these coupled motions may facilitate the greater total impulse required during acceleration compared with maximum velocity sprinting. Therefore, we believe there is some merit in promoting transverse shoulder plane motion, however, we have added a note before the ‘limitations’ section that coaches should consider on a case basis whether accentuated movement in both the sagittal and transverse planes is desired.

Lines 476-483: How do you propose this individual level analysis? Also, to be ecologically driven, wouldn’t it be best to analyze on the field during practice and require some type of sensor, such as an IMU?

We have highlighted the specific analyses that were implemented to evaluate individual-level changes. However, we would like to clarify whether the reviewer is asking how we would propose individual level analyses be carried out by coaches in the field. We would also like to clarify the comment regarding ‘ecologically driven’ analysis. While we agree that for assessments to be ecologically valid they should be conducted on field during practice, we do not discount the value of performing assessments in less representative environments to facilitate adequate data collection.

The information from Lines 503-508 needs to be woven into the introduction as well. It will help drive home the point that adaptability is good and leads to higher performance.

A short section in the introduction has been added to convey that movement variability afforded by WR may be practically useful in training to develop adaptability, and that adaptability facilitates task execution in a wider array of scenarios. This is applicable for field-based athletes who must negotiate dynamic and changing constraints during match play.

Consider making mention of adaptability in the context of injury prevention, not just performance.

We have amended the section under the subheading ‘skill acquisition and coaching implications’ to include mention of the proposed functional role of adaptability in injury prevention.

In the limitations section, make mention of the study not being ecological valid because data was collected in a laboratory, not on the playing field in a game or practice environment.

As with the previous comment regarding ecological validity; while we make mention of skill acquisition approaches grounded in complex systems theory, we do not explicitly refer to ecological dynamics. We predominantly refer to constraints on the movement system, or constraints as movement control parameters, which we don’t necessarily feel have to be implemented in a representative environment if the goal is to increase action capabilities. Nevertheless, we have added a statement in the limitations section noting that because complex systems-based pedagogical approaches emphasise ecological validity in training, it is reasonable to propose that testing should occur in an ecologically valid setting as well.

Conclusions
Good section, clean advice at the end.

We thank the reviewer for their positive feedback on this section.

Notes on Figures

Beautiful figures overall. One note, please bold the axes units and titles for figure 7 (joint kinematic data).

Axes units and titles have been made bold for greater clarity in Figure 7.

Minor Comments

Introduction:
Line 74: “…often utilized deconstructed, part practice drills...” Something is missing, or the punctuation is in the wrong place. Or deconstructed what?

We have rephrased this to simply ‘skill deconstruction’, and hope this conveys more clearly the approach traditionally taken by strength and conditioning coaches in developing sprint technique.

Line 79-80: “…by coaches to afford specific movement…”, consider changing “afford” to “influence”.

Change has been made in line with reviewer suggestion.

Line 81: “Both approaches” should be spelled out because I am not sure which two approaches you are referring to. I am guessing deconstructed and constraint, but I am certain.

We have sought to clarify that the two approaches being referred to are (i) the use of constraints to shape movement in a particular direction in line with a desired movement outcome, and (ii) the use of constraints to induce variability generally and facilitate implicit exploration of movement patterns.

Line 88: Remove “particular” because the segments are given after the comma.

Change has been made in line with reviewer suggestion.

Line 91: Remove “particular”

Change has been made in line with reviewer suggestion.

Line 92: “…are seen as being indicative of overload having occurred [].” Consider changing to “…indicate overload has occurred [].”

Change has been made in line with reviewer suggestion.

Methods:
Line 114: Include the “.0” for “72” to stay consistent with the other means.

Change has been made in line with reviewer suggestion.

Lines 118-120: How was that average distance and average running speed determined for these players?

While no direct measurement of the study participants was performed, the range provided is based on available literature relating to semi-professional Australian Rules football players in-season. The relevant citations have been added here.

Line 221: Add “cubic” in front of “spline” which I assume was the polynomial spline used.

Change has been made in line with reviewer suggestion.

Lines 228-229: Indicate if a researcher identified these points or if the V3D kinematic algorithm did.

We have clarified that an event detection algorithm in Visual 3D set to identify the initial increase in toe marker vertical displacement was used to identify toe-off timepoints.

Results:
Lines 361-362: “…, except for in the heavy anterior shank condition…” does not fit with the sentence and should be deleted.

Change has been made in line with reviewer suggestion.

Line 439: Include a comma between “condition pelvic”

Comma is now included as per reviewer’s suggestion.

Discussion:
Lines 506-515: Condense this section, it is superfluous, especially the two sentences from 511-515.

The authors agree with this feedback and this section has been condensed accordingly.

1.	Freeman BW, Talpey SW, James LP, Rayner RJ, Young WB. Preseason and in-season high-speed running demands of 2 professional Australian Rules football teams. Sports Health. 2024:19417381241265114. Epub 20240822. doi: 10.1177/19417381241265114. PubMed PMID: 39171493.

2.	Caldwell BP, Peters DM. Seasonal variation in physiological fitness of a semiprofessional soccer team. J Strength Cond Res. 2009;23(5):1370-7. doi: 10.1519/JSC.0b013e3181a4e82f. PubMed PMID: 19620929.

3.	Evangelos B, Lefteris M, Aristotelis G, Ioannis G, Natalia K. Aerobic and anaerobic capacity of professional soccer players in annual macrocycle. J Phys Educ Sport. 2016;16(2):527.

4.	Ostojic SM. Seasonal alterations in body composition and sprint performance of elite soccer players. J Exerc Physiol. 2003;6(3):11-4.

5.	Nakamoto H, Ishii Y, Ikudome S, Ohta Y. Kinesthetic aftereffects induced by a weighted tool on movement correction in baseball batting. Hum Mov Sci. 2012;31(6):1529-40. Epub 2012/06/16. doi: 10.1016/j.humov.2012.04.005. PubMed PMID: 22698835.

6.	Ohta Y, Ishii Y, Ikudome S, Nakamoto H. Warm-up with weighted bat and adjustment of upper limb muscle activity in bat swinging under movement correction conditions. Percept Mot Skills. 2014;118(1):96-113. doi: 10.2466/23.25.PMS.118k15w8. PubMed PMID: 24724516.

7.	Slawinski J, Bonnefoy A, Ontanon G, Lévêque J-M, Miller C, Riquet A, et al. Segment-interaction in sprint start: Analysis of 3D angular velocity and kinetic energy in elite sprinters. J Biomech. 2010;43(8):1494-502.
